# Comparative Survey of People with ME/CFS in Italy, Latvia, and the UK: A Report on Behalf of the Socioeconomics Working Group of the European ME/CFS Research Network (EUROMENE)

**DOI:** 10.3390/medicina57030300

**Published:** 2021-03-23

**Authors:** Elenka Brenna, Diana Araja, Derek F. H. Pheby

**Affiliations:** 1 Department of Economics and Finance, Università Cattolica del Sacro Cuore, Largo Agostino Gemelli 1, 20123 Milan, Italy; 2 Department of Dosage Form Technology, Faculty of Pharmacy, Institute of Microbiology and Virology, Riga Stradins University, Dzirciema Street 16, LV-1007 Riga, Latvia; diana.araja@rsu.lv; 3 Society and Health, Buckinghamshire New University, High Wycombe HP11 2JZ, UK; derekpheby@btinternet.com

**Keywords:** Myalgic Encephalomyelitis, chronic fatigue syndrome, ME/CFS, economic impact, medical care, social care, quality of life

## Abstract

*Background and Objectives*: A comparative survey of Myalgic Encephalomyelitis/Chronic Fatigue Syndrome (ME/CFS) patients was carried out in three countries, with the aim of identifying appropriate policy measures designed to alleviate the burden of disease both on patients and their families, and also on public institutions. The survey addressed demographic features, the economic impact of the disease on household incomes, patterns of medical and social care, specific therapies, social relationships, and the impact of the illness on quality of life. *Materials and Methods*: Parallel surveys were undertaken in Italy, Latvia, and the UK. There were 88 completed responses from Italy, 75 from Latvia, and 448 from the UK. To facilitate comparisons, 95% confidence intervals were calculated in respect of responses to questions from all three countries. To explore to what extent general practitioners (GPs) manage ME/CFS disease, a separate questionnaire for GPs, with questions about the criteria for granting a diagnosis, laboratory examinations, the involvement of specialists, and methods of treatment, was undertaken in Latvia, and there were 91 completed responses from GPs. *Results*: The results are presented in respect of sociodemographic information, household income, disease progression and management, perceived effectiveness of treatment, responsibility for medical care, personal care, difficulty explaining the illness, and quality of life. Demographic details were similar in all three countries, and the impact of illness on net household incomes and quality of life. There were significant differences between the three countries in illness progression and management, which may reflect differences in patterns of health care and in societal attitudes. Graded exercise therapy, practiced in the UK, was found to be universally ineffective. *Conclusions*: There were similarities between respondents in all three countries in terms of demographic features, the impact of the illness on household incomes and on quality of life, and on difficulties experienced by respondents in discussing their illness with doctors, but also differences in patterns of medical care, availability of social care, and societal attitudes to ME/CFS.

## 1. Introduction

Myalgic Encephalomyelitis/Chronic Fatigue Syndrome (ME/CFS) is a complex, multi-system disorder, with severe, profound incapacitating fatigue not alleviated by rest, and post-exertional malaise. Other symptoms include cognitive dysfunction, sleep disturbance, and muscle pain. As a result, marked reductions in functional activity and quality of life are encountered [1]. Cases vary markedly in the symptoms they manifest, in severity, and in disease progression. ME/CFS most frequently occurs between ages 20 and 50 but can affect all ages. The majority of patients are female [2]. UK experience suggests that there may be two million patients throughout Europe [3]. Much work has been carried out over several decades to investigate the nature of the syndrome, but marked uncertainty remains over its definition, diagnosis, treatment, and economic impact [4].

The problem of determining the economic impact of ME/CFS in Europe was considered by the socioeconomics working group of EUROMENE (*vide infra*). The economic burden is significant, with productivity losses appearing to be the largest cost element, while effective prevention and treatment give scope for substantial cost reductions. There are problems of economic evaluation because of the arbitrariness of case definitions, and doctors who are unable to diagnose the condition, for reasons including disbelief and lack of understanding, so there is a lack of accurate incidence and prevalence data. Recommendations of the working group include the use of the Fukuda (CDC-1994) case definition and Canadian Consensus Criteria (CCC), a pan-European common symptom checklist, implementation of prevalence-based cost-of-illness studies in different countries using an agreed data list, the use of purchasing power parities (PPP) to facilitate international comparisons, and the use of EuroQol-5D to measure health status [5].

The European Network on ME/CFS (EUROMENE) is a collaborative, Europe-wide, consortium aiming to address serious gaps in knowledge of ME/CFS. In 2016, EUROMENE received funding from the European Union through the COST programme, and was formally constituted as COST Action 15111. This action aims to “promote further research on ME/CFS with high economic impact” [6]. Working Group 3, on socioeconomics, has endeavored to appraise the economic implications of the disease, its specific objectives including surveying data from European countries on the economic losses due to ME/CFS and developing ways to calculate the direct and indirect economic burdens due to ME/CFS [7].

In pursuit of these objectives, a comparative questionnaire study of ME/CFS patients was carried out in three countries, Italy, Latvia, and the UK, with the aim of identifying appropriate policy measures designed to alleviate the burden of disease both on patients and their families, and on governments. In particular, reducing diagnostic delays should limit progression to severe, prolonged disease, with consequent reductions in its economic impact, including direct and indirect costs, and, most importantly, productivity costs.

## 2. Methods

Parallel surveys were undertaken in Italy, Latvia, and the UK. In Italy, a questionnaire (Supplementary Material) was distributed to 104 adult patients living in the north of the country, with the support of the Association of Patients CFS Onlus, which has an important role in assisting and supporting medical research and in disseminating knowledge of the disease [8]. The questionnaire had several sections. The first section sought general information (age, gender, education, place of residence, etc.), the second section addressed clinical history, and the third focused on the socio-economic consequences of the disease, including restrictions on daily life, sources of assistance, and understanding and awareness of the disease. The final section sought information on health status, reliance on physicians, the possible causes of illness, and future expectations. Quality of life was assessed using the instrument EuroQol-5D [9,10,11]. The patients were also asked to rate their quality of life in a scale from 0 to 100, where 100 represents the best imaginable QoL and 0 the worst, for the year before onset of illness, and for the year immediately preceding completion of the survey.

In Latvia, the patients’ questionnaire (Supplementary Material) has been designed in accordance with the questionnaire prepared by the Italian team of Working Group 3 (socioeconomic), employed in the European program COST Action 15111 EUROMENE, in order to get a comparable data. The sample has included 75 valid observations, performed by 62 women and 13 men. Simultaneously, a questionnaire for GPs was distributed with support of the Latvian Association of Rural Family Doctors, taking into account that this association represents GPs working in urban and rural areas. The survey had included 20 questions, mostly on the criteria for granting a diagnosis, laboratory examinations, the involvement of specialists, and the methods of treatment. There were 91 completed responses from GPs.

To obtain comparison data from the UK, an internet survey was set up using the facility ‘Smartsurvey’. A link to the questionnaire was circulated on 19 October 2020 via the internet group ‘LocalME’, with a request for UK residents with medically diagnosed ME/CFS to respond by 31 October. 448 questionnaires were completed by the deadline. The survey was structured in order to replicate as much as possible the Italian original questionnaire, though some variations were inevitable because of differences in the ways in which healthcare services are delivered. For the international comparison report we have calculated 95% confidence intervals for most of the parameters examined.

## 3. Results

In Italy, 88 questionnaires were correctly completed, and in Latvia, there were 75 valid responses from patients and 91 completed responses from GPs. There were 448 completed responses to the UK survey. Comparative results are presented below under the following headings—sociodemographic information, household income, disease progression and management, perceived effectiveness of treatment, responsibility for medical care, personal care, difficulty explaining the illness, and quality of life. The results of the additional GP survey in Latvia on the management of ME/CFS are provided in the concluding section.

### 3.1. Sociodemographic Information

The respondents ranged in age from 17 to 81, with an average of 50 years, and there was no significant difference between the three countries in terms of average age. In addition, there was no significant difference between the three countries in the gender distribution of respondents; in all three, a large majority of respondents were female. A very much higher proportion of UK respondents had post-school educational qualifications than in Latvia or Italy, but there was no significant difference between the latter two countries in that respect. Around half of all respondents in the three countries were married. In Latvia, a third of respondents lived alone. The proportions in Italy and the UK were lower, but these differences were not statistically significant. The results are detailed in Table 1.

### 3.2. Household Income

According to literature, the disease significantly impacts on the personal income, because patients are frequently unable to work. International comparisons are very difficult due to the heterogeneity of average income in UE countries. Looking at Eurostat [12], the mean equivalized net income in 2018 was respectively €19,208 (Italy), €25,642 (UK), and €8740 (Latvia). However, in Italy and the UK, only 44.1% and 54.6% respondents respectively declared an income higher than €15,000, which supports the hypothesis of an impact of the disease on individual productivity. In Latvia, no patients declared an income higher than €15,000, but this is due to the fact that income is relatively lower in this country. This international comparison is summarized in Table 2.

### 3.3. Disease Progression and Management

There were significant differences between the three countries in the proportions of respondents reporting having had more than ten investigations, having experienced more than ten symptoms, or having had more than five treatments. Italian respondents reported the most investigations and treatments and the most symptoms, which may be associated with a lack of appropriates guidelines for diagnosing ME/CFS, and the Latvians least, with the UK respondents occupying an intermediate position. UK respondents were significantly more likely than others to report that their symptoms fluctuated in severity (Table 3).

### 3.4. Perceived Effectiveness of Treatment

As regards the use of non-pharmacological treatments, in all three countries there were free text responses concerning the treatments followed during the last five years, in particular physiotherapy, cognitive behaviour therapy (CBT) and graded exercise therapy (GET). For all countries, physiotherapy is the most widespread treatment, with 41.7% Italian patients receiving it (or having received it in the last five years), 30.8% Latvian patients and fewer UK patients (14.3%). CBT was also reported by 23.5% Italians, 33.3% Latvians, and 7.0% UK participants.

A specific question asked if patients considered non-pharmacological treatments effective. Whilst in Italy only 8% patients answered positively, this percentage rises at 52% for Latvia and 57.7% for UK.

With regards to this item, it seems that Italian patients are not satisfied with the therapies received. A specific response regarding GET was obtained only from the UK, and it is noteworthy that only one respondent found it effective. These findings are detailed in Table 4.

### 3.5. Responsibility for Medical Care

GPs were significantly more likely to have primary responsibility for medical care in Latvia than in either Italy or the UK. This probably reflects the situation that there is not a specific patients’ organization for ME/CFS in Latvia, and the GP is a ‘gate-keeper’ for patients in diagnostic and treatment process. In Italy, the search for a correct diagnosis and the absence of appropriate guidelines for the disease identification pushes patients to ask for specialist consultations, as well as many diagnostic tests being performed before a final diagnosis is arrived at.

On the contrary, specialists had little involvement in the care of the UK patients. Other healthcare professionals were involved in the care of a higher proportion of UK respondents than was found in either Italy or Latvia. In the latter case, there was very little involvement of other professionals (see Table 5).

### 3.6. Personal Care

Significantly fewer Latvian respondents had family assistance with personal care than was found in either Italy or the UK. Other (non-family) sources of personal care assistance were reported by Italian or Latvian patients while nearly a fifth of UK respondents were cared for by non-family members. No external help with personal care was reported by nearly one in five of Italian respondents and a smaller proportion of UK ones. No response from Latvian patients. A large proportion of Italian and Latvian respondents reported that they had capacity for self-care, as well as a significantly smaller proportion, but still a majority, of UK respondents (Table 6).

### 3.7. Difficulty Explaining the Illness

One of the major difficulties for CFS/ME patients consists in explaining the symptoms. In Italy and the UK, around three-quarters of all respondents reported difficulty explaining their illnesses to physicians, but in Latvia only a quarter of respondents had this problem. This difference was statistically significant. When it came to explaining the illness to the family, though, nearly three-quarters of Italian respondents had trouble, while for Latvia and UK this figure decreases to less than 50%. Again, this difference was statistically significant. A quarter of Latvian respondents expressed difficulty explaining their illnesses to friends, compared with about two-thirds of British respondents and more than four-fifths of Italian ones. All these differences were statistically significant. The Latvians least frequently had difficulty explaining their illnesses to employers, compared with more than half of UK respondents and nearly two-thirds of Italians. However, these differences were not statistically significant (Table 7).

### 3.8. Quality of Life

In all three countries, a marked diminution in quality of life (scored 0 to 100) between the year before illness and the most recent year was reported. All these changes were statistically significant. The reported quality of life prior to illness was significantly higher in Italy than in the other countries and was lowest in Latvia. The diminution in perceived life quality as a result of illness was lowest in Latvia, where the mean quality of life score during illness was significantly higher than in either Italy or the UK. Indeed, it was in Italy where the greatest decline in average quality of life as a result of illness occurred (Table 8). To this extent, a study was carried out in Italy aimed at demonstrating the impact of selected variables on the probability of experiencing a decrease higher than 50 points in self-reported quality of life. It turned out, for example, that having more than 10 symptoms and being treated with more than 5 treatments was associated with this large reduction in quality of life [13].

### 3.9. Results of the Additional GP Survey in Latvia on the Management of CFS

In Latvia, there were 91 valid responses to the GP survey, which included questions on the criteria for making a diagnosis, laboratory examinations, referral to specialists and methods of treatment. For making the diagnosis, the results showed that 13 responders (14%) used the Fukuda case definition. 61 responders (67%) used the ICD-10 code R53 (malaise and fatigue), while 18 (20%) used code G93.3 (post viral fatigue syndrome). 5 respondents (5.5%) used ICD-10 code B94.8 (sequelae of other specified infectious and parasitic diseases). The multiplicity of codes used to record diagnoses of ME/CFS contributes to the problem of determining numbers of patients. Moreover, 70% of GPs reported that patients had difficulty in describing their symptoms. All the participating GPs used laboratory tests in the diagnostic process, some more than others, with 35 respondents (38%) using more than ten different tests. 65 GP respondents (71%) referred patients for specialist care and diagnostic support. Specialists referred to included neurologists (62%), psychiatrists or psychotherapists (30%), and infectious disease or other specialists (13%). 70% of GP respondents indicated the presence of comorbidities in their ME/CFS patients. GP management included medication, referral to physiotherapy, psychotherapy, osteopathy, homeopathy and lifestyle adjustment, but 59% of respondents regarded the disease as incurable. Improvements to the care of ME/CFS patients suggested by GP respondents included the development of a specialists’ consortium, better information for patients, public funding for psychotherapy consultations, additional training, and more time for conversation with patients.

## 4. Discussion

In the UK, treating physicians were not identified and therefore could not be interviewed. Similarly, in Italy recruitment was via a patients’ organization and did not involve treating physicians. In Latvia, participant selection was based on ICD-10 diagnoses. This was following advice from the country’s only secondary referral center for ME/CFS. The diversity of recruitment methods in the three countries was probably a source of strength rather than weakness, as the findings from the survey from the three countries revealed some very similar problems and concerns, suggesting that patients’ experiences were universal in nature, and not confined to any one country or health care system. It is interesting that, despite differences in health care systems, diagnostic methods, recruitment methods and survey media, there was a very considerable similarity in the experiences of respondents in all three countries.

Respondents in all three countries were similar in terms of average age and had the same preponderance of females. There were no significant differences in the proportions of respondents who were married or living alone, or who had post-school educational qualifications. In Latvia, the UK, and Italy, net household incomes were lower than the national average, indicating the impact of illness on incomes in all countries. As regards illness management and progression, there were significant differences between the three countries. Italians reported more symptoms, more investigations and more treatments than respondents from the other countries, and the Latvians the least. This may be due to the absence of appropriate guidelines for the management of the disease in Italy. We did not elicit any information on the nature of the investigations carried out, because the purpose of this question was to obtain a measure of the extent to which doctors in the three countries were taking seriously their patients’ illnesses and were actively working to investigate them.

Symptom fluctuation was significantly more marked among the UK respondents than among the others. It was noteworthy that graded exercise therapy, in the UK, was found to be universally ineffective, and none of the Italians reported having had this therapy. There were reported differences between the three countries in who had responsibility for providing medical care, but these may reflect differences in the management of the disease in each country. Thus, GPs more frequently had principal responsibility for medical care in Latvia than in Italy or the UK and this probably reflects the fact that in Latvia GPs perform the gate-keeper role for patients in the diagnostic and treatment process. Healthcare professionals other than doctors were more frequently involved in clinical care in the UK than in either Latvia or Italy, which is likely to be related to the pattern of delivery of primary care in the UK via the National Health Service.

In terms of personal care also, differences in response between the three countries were more likely to reflect differences in the way in which social care is delivered in the three countries. Thus, non-family care assistance was almost entirely confined to the UK, where capacity for self-care was less prevalent that in the other countries. Similarly, variations in difficulty explaining the disease to physicians was least widespread in Latvia, but this may be attributable to the fact that physicians there were centrally involved in the identification of potential respondents. Other variations, e.g., in explaining the illness to family, friends or employers, may reflect differences in society in the three countries. Italians were most likely to have trouble in explaining the illness to families, while Latvians had the least difficulty explaining the illness to friends or employers, which suggests that there may be greater understanding of the illness in the general population than is the case in either Italy or the UK.

While there were differences between the three countries in perceived quality of life both before and during illness, the trend was similar in Italy, Latvia, and the UK, with marked diminution in quality of life being reported in all three countries as a result of illness. In Latvia, the smaller gap in quality of life as a result of illness could be explained by a greater decrease in quality of life before diagnosis, with a subsequent smaller decrease in quality of life after diagnosis and initiation of treatment. While we do not wish to overinterpret these data, it is likely that the initial differences between the three countries reported prior to illness, for example in income and educational attainments, may reflect overall socioeconomic differences between them, while there is substantial convergence between all three countries in patient experience once illness becomes established.

The strength of this study is that this is the first study of ME/CFS patients conducted on a transnational, comparative basis in Europe. It demonstrates that the basic demographic features of the illness, in terms for example of the average age of participants and the gender distribution, are very similar in the three countries studied. Where the responses from the three countries differ, this largely reflects socioeconomic differences between countries, or differences in the way in which medical services are delivered. Although there was not a single template for the recruitment of patients, we endeavored to ensure the comparability of patients through the recruitment process. Thus, in Italy, only patients with a recognized diagnosis of CFS/ME were selected. Similarly, in Latvia, from almost 300 respondents to the on-line survey on CFS symptoms, only participants with ICD-10 diagnosis codes G93.3, R53 and B94.8 were involved in data analysis for the purposes of this paper, while in the UK patients with medically confirmed diagnoses of ME/CFS were self-selected via an internet-based patient support group.

The finding that ME/CFS has a substantial impact on net household income is consistent with the previous conclusion of the socioeconomics working group of EUROMENE that the economic impact of ME/CFS is substantial [5]. The reports of household income are worrying, given average 2018 incomes of €25,642 in the UK, €19,208 in Italy, and €8740 in Latvia [12]. International comparisons are very difficult due to the heterogeneity of average incomes in European countries, In Italy, 44.1% of respondents declared an income higher than €15,000, while the comparable proportion among UK respondents was 54.6% of respondents compared with 92% of the UK general population in 2017–18 [14]. In Latvia, annual incomes among the general population are lower than in Italy and the UK. No Latvian respondents reported an annual income per household member in excess of €15,000. Despite these differences between the three countries under consideration, the pattern is consistent; in all three countries, the respondents tended to report net incomes per household member which were substantially lower than those found among the general population. This underlines the negative impact of having ME/CFS on individual productivity and capacity to work and indicates that this impact is substantial. Our findings are consistent with other research demonstrating a very substantial impact of ME/CFS on productivity costs. Thus, Reynolds et al. analyzed data from a surveillance study of ME/CFS in Wichita, Kansas, and concluded that lost productivity due to ME/CFS was substantial both in absolute terms and in comparison with other major illnesses [15], while Collin et al. analyzed data from the UK CFS/ME National Outcomes Database, and concluded that ME/CFS causes huge productivity costs amongst the small fraction of adults with ME/CFS who access specialist services [16].

The paper did not address the relationship between severity and economic impacts, the importance of which we emphasized in a previous paper [5]. It is likely, though, that productivity costs were higher among the more severely ill patients, because being housebound or bedbound, severely ill patients are generally unable to work at all. Health care system costs are more complicated, because many severely ill patients receive no support or help from the health care system at all, due to the failure of primary care physicians to diagnose the illness. Such failure is widespread, with evidence that between a third to a half of all GPs, over several decades and a variety of geographical locations, expressing disbelief or failing to recognize ME/CFS as a genuine clinical entity [17,18].

This is consistent with the finding that a large proportion of respondents, particularly in Italy and the UK, have difficulty explaining their illness to doctors, as reported by the working group’s literature review of knowledge and understanding of ME/CFS among GPs [17], which established that disbelief and lack of knowledge were widespread in primary care, while our survey of perceptions of GP knowledge and understanding among EUROMENE participants suggests that this problem exists throughout Europe [18].

This study initiative aimed to identify policy measures designed to alleviate the burden of disease on patients and their families, and on governments, in particular by reducing delays in diagnosis, and has enabled certain changes in the way in which services are delivered to be identified. Thus, in Latvia, the survey was paralleled by a survey of GPs, in which a number of improvements were suggested, including establishment of a consortium of specialists, the creation and use of clinical algorithms and patient pathways, better information for patients, reimbursement from public funds of psychotherapists’ consultation fees, additional training, and more time for patient consultations. Another possible improvement recommended from Latvia was the establishment of a disease register, which would facilitate disease management by GPs, the development of patient pathways, and improved disease monitoring.

## 5. Conclusions

This comparative survey of ME/CFS patients in Italy, Latvia, and the UK has demonstrated marked similarities between respondents in all three countries in terms of demographic features, the impact of the illness on household incomes and on quality of life, and on difficulties experienced by respondents in discussing their illness with doctors. There were differences in terms of patterns of medical care, the availability of social care, and societal attitudes to ME/CFS. There is a need for internationally shared protocols for the disease treatment and diagnosis. More empirical research is required in Europe on ME/CFS patients’ needs in order to develop adequate care pathways.

## Figures and Tables

**Table 1:** Sociodemographic data.

Item	Country	No. Respondents	Mean	Standard Deviation (SD)	No. Responding ‘Yes’	%	95% Confidence Interval (%)
Age (years)	Italy	88	47.0	13.9			44.1–49.4
	Latvia	75	50.0	14.7			46.6–53.3
	UK	447	42.1	14.0			40.9–43.5
Gender (No. females)	Italy	88			68	77.3	67.1–87.4
	Latvia	75			62	82.7	73.1–92.3
	UK	385			332	86.3	82.5–90.0
Education (No. with post-school qualifications)	Italy	88			34	38.7	21.9–55.3
	Latvia	74			32	43.2	25.7–60.8
	UK	374			314	83.9	79.8–88.1
Marital status (No. married)	Italy	88			39	44.3	28.4–60.2
	Latvia	75			45	60.0	45.4–74.6
	UK	446			205	46.0	39.0–52.9
No. living alone	Italy	88			17	19.4	0.2–38.5
	Latvia	74			25	33.8	14.9–52.7
	UK	447			107	23.9	15.7–32.2

**Table 2:** Household incomes.

Item	Country	No. Respondents	No. Responding ‘Yes’	%	95% Confidence Interval (%)
Household income (No. with > €15,000 p.a.)	Italy	85	38	44.1	28.6–60.8
Household income, per member (No. with > €15,000 p.a.)	Latvia	65	0	0.0	-
	UK	443	242	54.6	48.2–61.0

**Table 3:** Disease progression and management.

Item	Country	No. Respondents	Mean	SD	No. Responding ‘Yes’	%	95% Confidence Interval (%)
No. symptoms	Italy	88	11.0	3.0			10.4–11.6
	Latvia	75	7.5	2.5			6.9–8.1
	UK	445	8.0	4.1			7.6–8.4
Treatments:							
- All	Italy	88	0.5	0.2			0.33–0.67
	Latvia	75	2.5	1.2			2.2–2.8
	UK	425	3.0	3.0			2.7–3.3
- Drug treatment	Italy	88	0.5	0.2			0.33–0.67
	Latvia	75	2.3	1.3			2.0–2.6
	UK	425	1.3	4.0			0.9–1.7
- Non-drug treatment	Italy	-	-	-			-
	Latvia	75	1.2	1.0			1.0–1.4
	UK	425	1.7	2.1			1.5–1.9
No. investigations	Italy	88	10.4	2.4			9.9–10.9
	Latvia	75	5.7	3.2			5.0–6.4
	UK	178	12.8	29.0			8.5–17.0
No. respondents reporting:							
>10 investigations	Italy	88			65	73.9	63.0–84.8
	Latvia	75			3	4.0	0.0–26.6
	UK	178			86	48.3	37.5–59.1
>10 symptoms	Italy	88			64	72.7	
	Latvia	75			7	9.3	4.6–18.0
	UK	445			86	19.3	1.8–27.8
>5 treatments	Italy	88			29	32.9	24.0–43.3
	Latvia	75			0	0.0	-
	UK	425			63	14.8	5.9–23.8
Variability of symptoms	Italy	86			48	55.8	41.5–70.1
	Latvia	75			53	70.7	58.2–83.2
	UK	446			410	91.9	89.2–94.6

**Table 4:** Perceived effectiveness of treatment.

Item	Country	No. Respondents	No. Responding ‘Yes’	%	95% Confidence Interval (%)
Treatments practiced in the last five years					
Physiotherapy	Italy	84	35	41.7	31.7–52.9
	Latvia	39	12	30.8	18.6–46.4
	UK	28	4	14.3	0.0–49.3
Cognitive behaviour therapy (CBT)	Italy	85	20	23.5	15.8–34.2
	Latvia	39	13	33.3	20.3 = 49.0
	UK	115	8	7.0	0.0–24.9
Graded exercise therapy (GET)	Italy	-	-	-	-
	Latvia	-	-	-	-
	UK	70	1	1.4	0.0–25.2
Perceived effectiveness of non-pharmacological treatments (No. finding treatment effective):	Italy	88	7	8.0	0.0–28.4
	Latvia	75	39	52.0	36.0–68.0
	UK	356	204	57.3	50.4–64.2

**Table 5:** Responsibility for medical care.

Item	Country	No. Respondents	No. Responding ‘Yes’	%	95% Confidence Interval (%)
Responsibility for medical care:					
- GPs	Italy	85	41	48.2	32.6–63.8
	Latvia	75	57	76.0	64.7–87.3
	UK	446	189	42.4	35.2–49.6
- Specialists	Italy	85	67	78.8	68.8–88.8
	Latvia	75	44	58.7	43.8–75.5
	UK	446	35	7.9	0.0–16.9
- Other	Italy	84	18	21.4	2.1–40.8
	Latvia	75	5	6.7	0.0–29.0
	UK	446	62	35.9	5.1–22.7

**Table 6:** Personal care.

Item	Country	No. Respondents	No. Responding ‘Yes’	%	95% Confidence Interval (%)
Assistance with Personal Care:					
- family	Italy	85	70	82.3	73.2–91.5
	Latvia	72	17	23.6	11.0–24.9
	UK	413	293	70.9	65.6–76.2
- others	Italy	85	1	1.2	0.0–22.7
	Latvia	72	0	0.0	-
	UK	413	76	18.4	9.5–27.3
- No-one	Italy	85	16	18.8	0.0–38.4
	Latvia	72	0	0.0	-
	UK	413	31	7.5	0.0–17.0
Capacity for self-care (number responding ‘Yes’)	Italy	84	73	86.9	79.0–94.8
	Latvia	72	58	77.3	70.2–90.9
	UK	443	253	57.1	50.9–63.3

**Table 7:** Difficulty explaining the illness.

Item	Country	No. Respondents	No. Responding ‘Yes’	%	95% Confidence Interval (%)
Difficulty explaining illness to:					
- Physicians	Italy	86	63	73.2	62.1–84.4
	Latvia	75	20	26.7	18.0–37.6
	UK	444	343	77.3	72.7–81.8
Family	Italy	84	60	71.4	59.8–83.1
	Latvia	75	35	46.7	35.8–57.9
	UK	446	222	49.8	43.1–56.5
Friends	Italy	83	68	81.9	72.6–91.3
	Latvia	75	20	26.7	18.0–37.6
	UK	444	290	65.3	59.7–70.9
Employers	Italy	76	49	64.5	50.8–78.1
	Latvia	75	30	40.0	29.7–51.3
	UK	407	233	57.2	50.8–63.7

**Table 8:** Quality of life.

Item	Country	No. Respondents	Mean	SD	95% Confidence Interval (%)
Quality of life:					
- before illness	Italy	84	90.3	9.7	88.2–92.4
	Latvia	74	74.6	24.0	69.0–80.2
	UK	439	80.9	23.0	78.7–82.7
- in past year	Italy	84	34.6	20.8	30.1–39.1
	Latvia	74	57.3	16.3	53.5–61.1
	UK	440	31.5	19.8	29.6–33.1

## Data Availability

Initial tabulations of data from the UK are available upon request from the author. Data from Italy are available on request from the author. The aggregated data of Latvia surveys are available on request from the Riga Stradins University.

## Supplementary Materials

The following are available online at https://www.mdpi.com/1648-9144/57/3/300/s1.

## References

[1] Carruthers B.M., Jain A.K., De Meirleir K.L., Peterson D.L., Klimas N.G., Lerner A.M., Bested A.C., Flor-Henry P., Joshi P., Powles P. (2003). Myalgic encephalomyelitis/chronic fatigue syndrome: Clinical working case definition, diagnostic and treatment protocols. J. Chronic Fatigue Syndr..

[2] Pheby D., Lacerda E., Nacul L., Drachler M.D.L., Campion P., Howe A., Poland F., Curran M., Featherstone V., Fayyaz S. (2011). A Disease Register for ME/CFS: Report of a Pilot Study. BMC Res. Notes.

[3] Action for ME. https://actionforme.org.uk/what-is-me/introduction/.

[4] Brenna E., Gitto L. (2017). The economic burden of Chronic Fatigue Syndrome/Myalgic Encephalomyelitis (CFS/ME): An initial summary of the existing evidence and recommendations for further research. Eur. J. Pers. Cent. Healthc..

[5] Pheby D.F., Araja D., Berkis U., Brenna E., Cullinan J., De Korwin J.-D., Gitto L., A Hughes D., Hunter R.M., Trepel D. (2020). The Development of a Consistent Europe-Wide Approach to Investigating the Economic Impact of Myalgic Encephalomyelitis (ME/CFS): A Report from the European Network on ME/CFS (EUROMENE). Healthcare.

[6] EUROMENE. http://www.euromene.eu.

[7] Memorandum of Understanding of COST Action 15111—EUROMENE. http://www.cost.eu/actions/CA15111/#tabs/Name;overview.

[8] Ardino1 R.B., Lorusso L. (2018). La syndrome da affaticamento cronico/encefalomielite mialgica: Le caratteristiche della malattia e il ruolo dell’Associazione malati CFS [Chronic fatigue syndrome/myalgic encephalomyelitis: The characteristics of the disease and the role of the CFS Patients Association. Politiche Sanit..

[9] (1990). EuroQol Group EuroQol—A new facility for the measurement of health-related quality of life. Health Policy.

[10] Williams A. (1995). The Role of EuroQol Instrument in Qaly Calculations, No 130chedp.

[11] Dolan P. (1997). Modeling Valuations for EuroQol Health States. Med. Care.

[12] Eurostat https://appsso.eurostat.ec.europa.eu/nui/submitViewTableAction.doc.

[13] Brenna E., Gitto L. (2018). Sindrome da affaticamento cronico: Una prima analisi empirica per l’Italia. Politiche Sanit..

[14] The Survey of Personal Incomes. https://www.gov.uk/government/statistics/percentile-points-from-1-to-99-for-total-income-before-and-after-tax.

[15] Reynolds K.J., Vernon S.D., Bouchery E., Reeves W.C. (2004). The economic impact of chronic fatigue syndrome. Cost Eff. Resour. Alloc..

[16] Collin S.M., Database U.C.N.O., Crawley E., May M.T., Sterne J.A., Hollingworth W. (2011). The impact of CFS/ME on employment and productivity in the UK: A cross-sectional study based on the CFS/ME national outcomes database. BMC Health Serv. Res..

[17] Pheby D., Araja D., Berkis U., Brenna E., Cullinan J., de Korwin J.-D., Gitto L., Hughes D., Hunter R., Trepel D. (2020). A Literature Review of GP Knowledge and Understanding of ME/CFS: A Report from the Socioeconomic Working Group of the European Network on ME/CFS (EUROMENE). Medicina.

[18] Cullinan J., Pheby D., Araja D., Berkis U., Brenna E., de Korwin J.-D., Gitto L., Hughes D., Hunter R., Trepel D. (2021). Perceptions of European ME/CFS Experts Concerning Knowledge and Understanding of ME/CFS among Primary Care Physicians in Europe: A Report from the European ME/CFS Research Network (EUROMENE). Medicina.

