# Supplementary material for: Comparative Survey of People with ME/CFS in Italy, Latvia, and the UK: A Report on Behalf of the Socioeconomics Working Group of the European ME/CFS Research Network (EUROMENE)
Source: Medicina (Kaunas). 2021 Mar 23;57(3):300. doi: 10.3390/medicina57030300 (PMC8005199; doi:10.3390/medicina57030300)
Supplement: Supplementary file 1 [file medicina-57-00300-s001.zip › New folder/CFS_questionnaire_LV.pdf]

Anketa pacientam
"Hroniskais noguruma sindroms"

RĪGAS STRADIŅA UNIVERSITĀTE

Anketa ir sagatavota COST (*Cooperation in Science and Technology*) projekta EUROMENE zinātniskā tīkla ietvaros, kurā Rīgas Stradiņa universitāte pārstāv Latviju un kura izveidošanas mērķi ir saistīti ar Mialģiskā encefalomielīta/ Hroniskā noguruma sindroma (ME/HNS) padziļinātu izpēti un starpvalstu sadarbību vienotas pieejas izstrādāšanai ME/HNS diagnostikai un multidisciplināras ārstēšanas vadlīniju izstrādāšanai. Anketa ir izveidota, lai noskaidrotu HNS slimnieku zināšanas par HNS slimību, saņemto veselības aprūpi un problēmām, kas saistītas ar HNS ietekmi uz dzīves kvalitāti.

HNS raksturīgākie simptomi ir hronisks nogurums vismaz pusgada laikā, kas nav atvieglojams ar atpūtu; galvassāpes, muskuļu sāpes, palielināti limfmezgli, locītavu sāpes, kakla sāpes, atmiņas traucējumi, miega problēmas. Bieži vien šiem simptomiem pievienojas arī depresija, ja slimnieka stāvoklis ir smags un dzīves kvalitāte ir zema. HNS var izpausties arī kā centrālās nervu sistēmas, imūnsistēmas, šūnu metabolisma, sirds un asinsvadu darbības traucējumi.

Ja saskatāt, ka minētie simptomi Jums lielākā vai mazākā mērā ir raksturīgi, aicinām aizpildīt pievienoto anketu un ar savu dalību veicināt hroniskā noguruma sindroma pacientu veselības aprūpes uzlabošanu.

Anketas aizpildīšanai nepieciešamas aptuveni 20 minūtes.

1. **Kad slimība (noturīgi simptomi) ir sākusies (norādiet aptuveni gadu un mēnesi) ?**

   ____

2. **Kā Jūs vērtējat savu pašreizējo stāvokli – vai slimība ir akūtā, vai hroniskā fāzē ?**

   Akūtā ☐
   Hroniskā ☐

3. **Kādus simptomus novērojat ? (vairākas atbildes ir iespējamas)**

   Epizodisks nogurums ☐
   Noturīgs nogurums ☐
   Svārstīgs nogurums (*raksturojams ar svārstībām starp sliktākiem un labvēlīgākiem periodiem*) ☐
   Gripas simptomiem līdzīgs nespēks ☐
   Galvassāpes ☐
   Kakla sāpes ☐
   Muskuļu sāpes ☐
   Paaugstināta vai pazemināta ķermeņa vidējā temperatūra (*norādiet, kāda ir ķermeņa vidējā temperatūra*) ▭
   Svārstīgs asinsspiediens ☐
   Kuņģa-zarnu trakta traucējumi ☐
   Urinēšanas traucējumi ☐
   Palielināti limfmezgli ☐
   Koncentrēšanās grūtības ☐

Atmiņas traucējumi ☐
Psiholoģiskais diskomforts (trauksme) ☐
Nomākts garastāvoklis ☐
Miega traucējumi ☐
Citi simptomi (norādiet) ____

____

4. **Vai novērotie simptomi ir konstanti vai variē ?**

Konstanti ☐
Mainīgi ☐

5. **Vai Jums ir noteikta kāda no diagnozēm, kas ir saistītas ar Hroniskā noguruma sindromu (turpmāk – HNS diagnoze) ?**

ICD 10 kods G93.3 – nogurums pēc vīrusa infekcijas (mialģiskais encefalomielīts) ☐
ICD 10 kods R53 (īpaši 53.82) – patoloģisks nogurums un nespēks ☐
ICD 10 kods B94.8 – citu precizētu infekcijas un parazitāru slimību sekas ☐
Diagnoze nav noteikta Pārejiet pie 8.jautājuma

6. **Cik ilgs laiks ir pagājis no pirmo noturīgo simptomu parādīšanās līdz HNS diagnozes noteikšanai ?**

3 mēnešu laikā ☐
1 gada laikā ☐
> 1 gada ☐

7. **Kādas specialitātes ārsts ir noteicis Jums HNS diagnozi ?**

Ģimenes ārsts ☐
Neirologs ☐
Infektologs ☐
Cits speciālists (norādiet) ____

8. **Vai Jums ir veiktas kādas medicīniskās pārbaudes saistībā ar HNS ?**

Jā ☐
Nē ☐

Ja atbilde ir "Jā", norādiet, kādas (vairākas atbildes ir iespējamas):

Standarta laboratoriskie izmeklējumi ☐
Infekcijas testi ☐
Imunoloģiskie testi ☐
Iekaisuma testi ☐
Vairogdziedzera pārbaudes ☐
Radioloģiskie izmeklējumi ☐
Kardioloģiskie izmeklējumi ☐
Gastroenteroloģiskie izmeklējumi ☐
Pulmonoloģiskie izmeklējumi ☐
Endokrinoloģiskie izmeklējumi ☐
Psihiatriskie izmeklējumi ☐
Neiroloģiskie izmeklējumi ☐

Cita veida izmeklējumi (norādiet) ____

____

9. **Vai Jums ir noteikta kāda ar HNS nesaistītā hroniskā saslimšana ?**

Jā ☐ Nē ☐

Ja atbilde ir "Jā", vēlams norādīt konkrētas diagnozes:

____

____

10. **Vai Jūs regulāri apmeklējat kādu ārstu saistībā ar HNS ? norādiet, kādu:**

Neapmeklēju ☐

Apmeklēju: ☐

	**Reizi mēnesī**	**Reizi ceturksnī**	**Reizi gadā**	**Reizi 2 gados**	**Retāk kā reizi 2 gados**
Ģimenes ārsts					
Neirologs					
Infektologs					
Cits speciālists _________________					

11. **Kādas metodes Jūs izmantojat HNS seku mazināšanai ? (vairākas atbildes ir iespējamas)**

Vispāratzītā (alopātiskā) medicīna ☐
Homeopātija ☐
Osteopātija ☐
Psihoterapija ☐
Fitoterapija ☐
Cita metode (norādiet) ____

12. **Kādas metodes (un preparātus) Jūs izmantojas HNS akūtajos stāvokļos ? (vairākas atbildes ir iespējamas)**

Recepšu zāles ☐
Bezrecepšu zāles ☐
Uztura bagātinātāji ☐
Fizioterapija ☐
Osteopātija ☐
Homeopātija ☐
Psihoterapija ☐
Cita metode (norādiet) ____

13. **Kura no metodēm (vai metožu kombinācijām), Jūsuprāt, deva labāku rezultātu? (norādiet)**

____

14. **Vai lietojat kādus medikamentus saistībā ar HNS ? (vairākas grupas ir iespējamas):**

Pretiekaisuma ☐
Pretsāpju ☐
Pretvīrusu ☐
Nomierinošie ☐
Antidepresanti ☐
Vitamīni un minerālvielas ☐
Citi (norādiet) ____
Nelietoju ☐

15. **Norādiet vidējās izmaksas mēnesī (*euro*), cik tērējat HNS ārstēšanai un negatīvu seku mazināšanai**

Zāles ☐ euro
Uztura bagātinātāji ☐ euro
Konvenciālās medicīnas veselības aprūpes pakalpojumi (vizītes pie ārstiem) ☐ euro
Homeopātija ☐ euro
Osteopātija ☐ euro
Psihoterapija ☐ euro
Fitoterapija ☐ euro
Cita metode (norādiet) ____ ☐ euro

16. **Ja Jūs zaudējat darba spējas HNS saasinājuma fāzē un nevarat apmeklēt darbu, norādiet, cik vidēji darba dienas tiek zaudētas noteiktajā laika periodā (piemēram, mēnesī)**

____

17. **Vai darba nespējas laikā Jūs aprūpējat sevi patstāvīgi, vai ar kāda palīdzību ?**

Patstāvīgi ☐
Ģimenes locekļi ☐
Aprūpētājs ☐
Cita alternatīva (norādiet) ____

18. **Atzīmējiet, ja Jums sagādā grūtības izskaidrot ar HNS saistītos simptomus, izjūtas un sekas (vairākas atbildes ir iespējamas):**

Ārstam ☐
Ģimenes locekļiem (radiniekiem) ☐
Draugiem ☐
Darba devējam (un kolēģiem) ☐

19. **Norādiet, kādas ikdienas aktivitātes sagādā grūtības saistībā ar HNS (1.kolonnā) un kāds ir Jūsu pašreizējais pašvērtējums – no 1 līdz 10 (cik augsti novērtējat pašreizējās spējas un stāvokli konkrētajā jomā: 1 – zemākais līmenis, 10 – augstākais līmenis) (2.kolonnā)**

Darbs (arī mācības un ģimenes aprūpe) ☐ ☐
Pašaprūpe ☐ ☐
Mobilitāte ☐ ☐
Emocionālais stāvoklis ☐ ☐
Fiziskais stāvoklis ☐ ☐

20. **Novērtējiet ar atzīmi no 1 (zemākais līmenis) līdz 10 (augstākais līmenis) savu ar veselības stāvokli saistīto dzīves kvalitāti pēdējā gada laikā** ☐

21. **Novērtējiet ar atzīmi no 1 (zemākais līmenis) līdz 10 (augstākais līmenis) savu ar veselības stāvokli saistīto dzīves kvalitāti pirms HNS konstatācijas** ☐

22. **Kā Jūs vērtējat savas saslimšanas smaguma pakāpi ?**

Viegla ☐
Vidēja ☐
Smaga ☐

23. **Kuri no HNS simptomiem, Jūsuprāt, ir vissmagākie ? (norādiet)**

____

24. **Kādi, Jūsuprāt, ir HNS galvenie cēloņi ? (vairākas atbildes ir iespējamas)**

Emocionālais stāvoklis ☐
Darba apstākļi ☐
Ģenētiskā pārmantojamība ☐
Autoimūnā saslimšana ☐
Infekcijas slimība ☐
Vides faktori ☐
Citi faktori (norādiet) ____

25. **Vai, Jūsuprāt, HNS simptomi tuvākajā nākotnē var izraisīt negatīvas izmaiņas Jūsu dzīves sfērās (vairākas atbildes ir iespējamas)**

Darbā, profesionālajā karjerā ☐
Ikdienas aktivitāšu veikšanā ☐
Fiziskajā veselībā ☐
Mentālajā veselībā ☐
Citos aspektos (norādiet) ____

26. **Vai Jūs saskatāt iespējas pilnīgi izārstēties no HNS ?**

Jā ☐
Nē ☐

27. **Vai Jūs veicat kādus profilaktiskus un veselības uzlabošanas pasākumus ? (vairākas atbildes ir iespējamas)**

Regulāras sporta nodarbības ☐
Atteikšanās no smēķēšanas un citiem kaitīgiem ieradumiem ☐
Veselīgs uzturs ☐
Psiholoģisko noturību veicinošās prakses ☐
Harmonizēts darba un atpūtas režīms ☐
Fizioloģiskā miega režīma ievērošana ☐
Citi pasākumi (norādiet) ____

Lūgums norādīt informāciju par sevi (Jūsu dati tiks izmantoti tikai apkopotā veidā pētījuma mērķiem)

28. **Jūsu dzimšanas gads** ☐

29. **Dzimums**

Sieviete ☐
Vīrietis ☐

30. **Izglītība**

Pamata ☐
Vidējā ☐
Vidējā profesionālā ☐
Augstākā ☐

31. Pamatnodarbošanās

Uzņēmējs ☐
Algotais darbinieks ☐
Pašnodarbinātā persona ☐
Students ☐
Nestrādājošais pensionārs ☐
Mājsaimniece ☐
Cits ____

32. Ģimenes stāvoklis

Precējies (-usies) ☐
Neprecējies (-usies) ☐
Atraitnis (-e) ☐
Šķīries (-usies) ☐

33. Mājsaimniecība

Patstāvīga ☐
Kopīga ar ģimenes locekļiem ☐
Cits variants ____

34. Bērnu skaits Jūsu ģimenē ☐

35. Jūsu vidējie ienākumi mēnesī (pēc nodokļu atskaitīšanas) uz vienu ģimenes locekli vai kopīgas mājsaimniecības locekli ☐ euro

36. Dzīves vieta (atzīmējiet vienu pozīciju katrā kolonnā)

Rīga vai Pierīga	☐	Apdzīvotā vieta (AV) līdz 2000 iedzīvotāju	☐
Vidzemes reģions	☐	AV no 2001 līdz 4000 iedzīvotāju	☐
Kurzemes reģions	☐	AV no 4001 līdz 10000 iedzīvotāju	☐
Zemgales reģions	☐	AV no 10001 līdz 50000 iedzīvotāju	☐
Latgales reģions	☐	AV ar iedzīvotāju skaitu virs 50000	☐

37. Jūsu ierosinājumi HNS pacientu veselības aprūpes uzlabošanai

____
____
____
____
____
____
____

Pateicamies par veltīto laiku un Jūsu ieguldījumu hroniskā noguruma sindroma pacientu veselības aprūpes uzlabošanā!
