# Supplementary material for: Comparative Survey of People with ME/CFS in Italy, Latvia, and the UK: A Report on Behalf of the Socioeconomics Working Group of the European ME/CFS Research Network (EUROMENE)
Source: Medicina (Kaunas). 2021 Mar 23;57(3):300. doi: 10.3390/medicina57030300 (PMC8005199; doi:10.3390/medicina57030300)
Supplement: Supplementary file 1 [file medicina-57-00300-s001.zip › New folder/UK questionnaire.pdf]

ME/CFS European Comparison Survey

Dear fellow members of the ME community,

I wonder if I could, please, request your assistance. I am working with European colleagues in the EUROMENE collaboration, and we have been very successful in stimulating research into ME/CFS throughout Europe.

Researchers in Latvia and Italy have conducted fairly small-scale epidemiological studies, with the intention of producing an international comparison, and would like to be able to compare their results with data from the UK, where most of the epidemiological research into ME/CFS in Europe has been undertaken.

I am very keen to encourage this development, as it is only through epidemiological research that we will be able to quantify the scale of the illness in Europe, which in turn will get added weight to our lobbying for increased resources both for research and for clinical and other services.

I have therefore developed a short survey instrument to enable collection of data which will facilitate the comparison my Latvian and Italian colleagues wish to make. This is necessarily limited in scope, because I am only asking the questions which have been asked in the surveys already carried out in Latvia and Italy. It's therefore quite short, and it should be possible to complete it in under ten minutes.

If you are a UK resident, have a medically confirmed diagnosis of ME/CFS, and would like to participate, you can find the survey by following this link:- https://www.smartsurvey.co.uk/s/GX4HMU/

Please feel free to send this link to anyone else who might be interested and may want to participate. I would be grateful for responses by the end of October, please.

The survey is of course completely anonymous, but, if you would like to receive a report of this study in due course and leave your email address at the end of the survey (or alternatively email me at derekpheby@btinternet.com), I will make sure that you that this is sent to you.

Yours faithfully,

Professor Derek Pheby (Chairman, Socioeconomics Working Group, EUROMENE).

No.	Question	Response
1	How old are you?	
	17 or younger	
	18-24	
	25-34	
	35-44	
	45-54	
	55-64	
	65 or older	
2	What is your gender?	
	Male	
	Female	
3	Do you have post-school educational qualifications?	
	Yes	
	No	

4	Are you married?	
	Yes	
	No	
5	Do you live alone?	
	Yes	
	No	
6	Does your net annual income (i.e. after tax) per household member exceed €15,000 (i.e. £13,650)?	
	Yes	
	No	
7	How many years and months have elapsed following the appearance of your first ME/CFS symptom?	
	[Free text]	
8	Are you housebound or bedbound as a result of having ME.CFS?	
	Yes	
	No	
9	How many investigations of your ME/CFS (blood tests, X-rays, scans, etc.) have you had?	
	[Free text]	
10	Who takes most responsibility for your clinical care?	
	GP	
	Hospital specialist	
	Other (please specify):	
	No-one	
11	Please list your main symptoms.	
	[Free text]	
12	Do your symptoms tend to fluctuate in severity over time?	
	Yes	
	No	
13	Please list any treatments you have had for ME/CFS,	

	[Free text]	
14	Please list any of the above treatments (if any) which were effective in relieving any of your symptoms	
	[Free text]	
15	Are you able to look after yourself?	
	Yes	
	No	
16	Who else assists you with your care?	
	Family member(s)	
	Statutory service(s)	
	Other (please specify):	
17	Quality of life: On a scale of 0 to 100, where 0 is the worst possible quality of life and 100 is the best possible, what average score of quality of life would you give for the year immediately before you fell ill?	
	[Free text]	
18	On the same scale, what score would you give for the past year?	
	[Free text]	
For the next four questions, please indicate whether you agree or disagree with the statement:-		
19	1) I find it difficult to discuss my illness with doctors	
	Strongly agree	
	Agree	
	Neither agree nor disagree	
	Disagree	
	Strongly disagree	
20	2) I find it difficult to discuss my illness with family members	
	Strongly agree	
	Agree	
	Neither agree nor disagree	

	Disagree	
	Strongly disagree	

21	3) I find it difficult to discuss my illness with friends	
	Strongly agree	
	Agree	
	Neither agree nor disagree	
	Disagree	
	Strongly disagree	
23	4) I find it difficult to discuss my illness with employers	
	Strongly agree	
	Agree	
	Neither agree nor disagree	
	Disagree	
	Strongly disagree	
24	Do you expect that at some stage you will fully recover from your illness?	
	Yes	
	No	
